# Identification and Growth Inhibitory Activity of the Chemical Constituents from *Imperata Cylindrica* Aerial Part Ethyl Acetate Extract

**DOI:** 10.3390/molecules23071807

**Published:** 2018-07-21

**Authors:** Yan Wang, James Zheng Shen, Yuk Wah Chan, Wing Shing Ho

**Affiliations:** School of Life Sciences, Chinese University of Hong Kong, Shatin, Hong Kong, China; 1155070144@link.cuhk.edu.hk (Y.W.); james_shen_zheng@alumni.cuhk.net (J.Z.S.); anthony.chan@link.cuhk.edu.hk (Y.W.C.)

**Keywords:** *Imperata cylindrica*, HPLC, ESI-MS/MS, growth inhibitory activity, cancer

## Abstract

*Imperata cylindrica* (L.) Raeusch. (IMP) aerial part ethyl acetate extract has anti-proliferative, pro-apoptotic, and pro-oxidative effects towards colorectal cancer in vitro. The chemical constituents of IMP aerial part ethyl acetate extract were isolated using high-performance liquid chromatography (HPLC) and identified with tandem mass spectrometry (ESI-MS/MS) in combination with ultraviolet-visible spectrophotometry and 400 MHz NMR. The growth inhibitory effects of each identified component on BT-549 (breast) and HT-29 (colon) cancer cell lines were evaluated after 48/72 h treatment by MTT assay. Four isolated compounds were identified as trans-p-Coumaric acid (**1**); 2-Methoxyestrone (**2**); 11, 16-Dihydroxypregn-4-ene-3, 20-dione (**3**); and Tricin (**4**). Compounds (**2**), (**3**), and (**4**) exhibited considerable growth inhibitory activities against BT-549 and HT-29 cancer cell lines. Compounds (**2**), (**3**), and (**4**) are potential candidates for novel anti-cancer agents against breast and colorectal cancers.

## 1. Introduction

*Imperata cylindrica* (L.) Raeusch. (IMP) is widely used for the treatment of hemorrhage, improvement of urination, and enhancement of the immune system [1]. Amounts of bio-active compounds isolated from IMP rhizomes and leaves were identified including benzoic acid and its derivatives [2], lignans [3], phenolic compounds [4], steroids [5], methoxylated flavonoids [6], and chromones [7].

Cancer is one of the leading causes of death worldwide. Herbal medicines are commonly used as both complementary ingredients and alternative therapies in cancer treatments. Potential bio-active components from herbal medicines can be isolated and purified using a high-performance liquid chromatography (HPLC) system. A tandem MS/MS detection system providing fragmentation information of the targets is one of the best choices adopted in chemical structural characterization and drug discovery [8,9].

Our previous study demonstrated that IMP aerial part ethyl acetate extract had growth-inhibiting, pro-apoptotic, and pro-oxidative effects on a colorectal cancer cell line HT-29 in vitro [10]. The present study aims to isolate the chemical constituents from IMP aerial part ethyl acetate extract and identify the bio-active compounds with considerable growth inhibitory activity against cancers.

## 2. Results

### 2.1. Isolation, Identification, and Quantification of Compounds *(**1**)–(**4**)*

**Figure 1:**
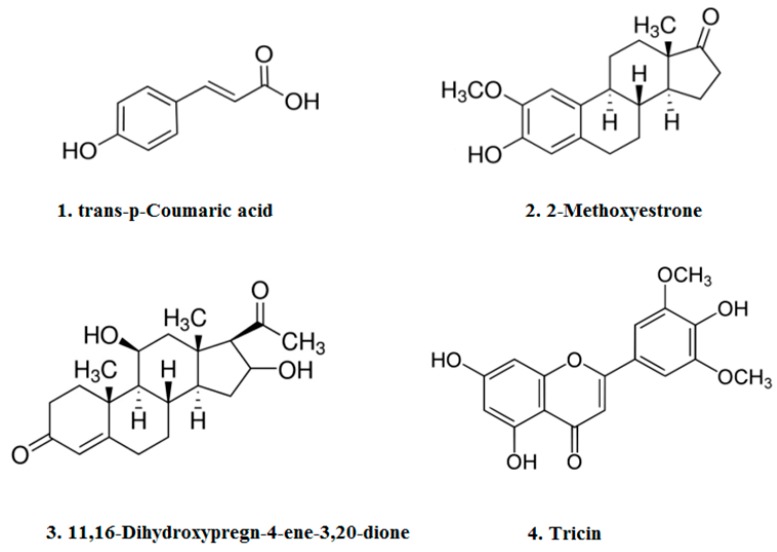
Chemical structures of compounds (**1**)–(**4**) isolated from IMP aerial part ethyl acetate extract. These are trans-p-Coumaric acid (**1**); 2-Methoxyestrone (**2**); 11, 16-Dihydroxypregn-4-ene-3, 20-dione (**3**); and Tricin (**4**).

Chemical structures of four compounds isolated from IMP aerial part ethyl acetate extract are shown in Figure 1.

#### 2.1.1. Compound (**1**): Trans-p-Coumaric Acid

**Figure 2:**
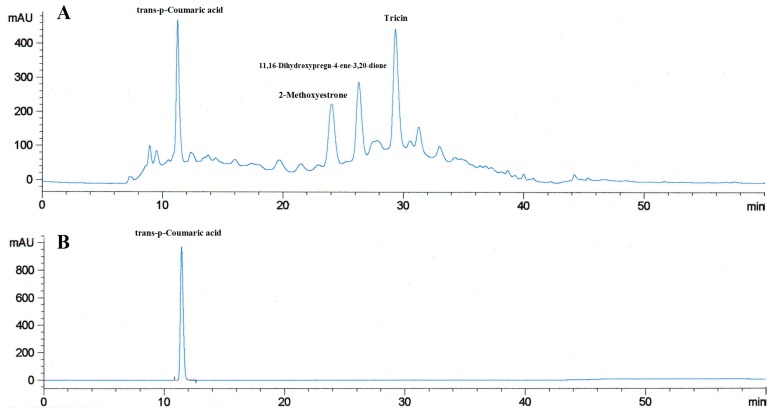
HPLC-DAD chromatogram of IMP aerial part ethyl acetate extract at 323 nm. IMP aerial part ethyl acetate extract solution (10 mg/mL in methanol, 20 µL) was analyzed in the 60 min HPLC gradient program. (**A**) The retention time of the trans-p-Coumaric acid standard (0.125 mg/mL in methanol, 20 µL) purchased from Sigma (**B**) was consistent with compound (**1**) in IMP aerial part ethyl acetate extract fingerprint.

The molecular formula of compound (**1**), C_9_H_8_O_3_, was identified by comparing the liquid chromatographic retention time, UV absorption spectrum, and ESI-MS/MS spectrum with the trans-p-Coumaric acid standard (Sigma Aldrich, St. Louis, MO, USA). The HPLC chromatogram of compound (**1**) and trans-p-Coumaric acid standard (in methanol) possessed the same identical retention time (Figure 2A,B).

**Figure 3:**
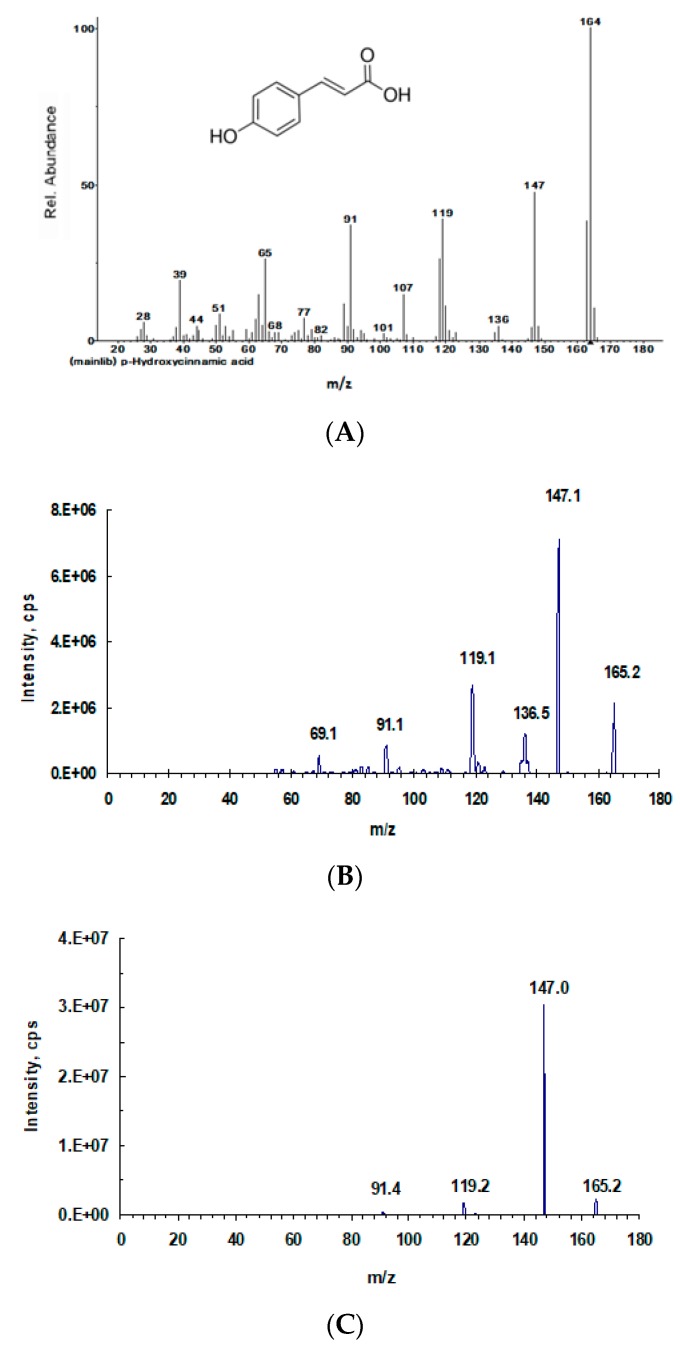
Relevant tandem mass (MS/MS) spectra. (**A**) trans-p-Coumaric acid spectrum (from NIST 14 mass spectral library); (**B**) isolated and purified compound (**1**); (**C**) trans-p-Coumaric acid standard (Sigma).

The MS/MS fragmentation pattern of compound (**1**) accurately matched with the MS^2^ spectrum from the NIST14 mass spectral database and the trans-p-Coumaric standard (Figure 3).

#### 2.1.2. Compound (**2**): 2-Methoxyestrone

**Figure 4:**
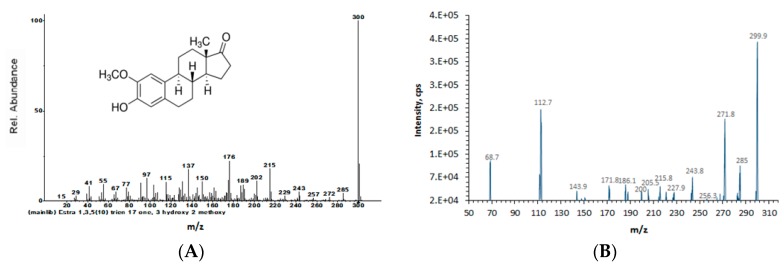
Relevant tandem mass (MS/MS) spectra. (**A**) 2-Methoxyestrone (from NIST 14 mass spectral library); (**B**) isolated and purified compound (**2**).

The molecular formula of compound (**2**), C_19_H_24_O_3_, was identified with the MS/MS spectrum by searching the NIST14 mass spectral database (Figure 4).

#### 2.1.3. Compound (**3**): 11, 16-Dihydroxypregn-4-ene-3, 20-dione

**Figure 5:**
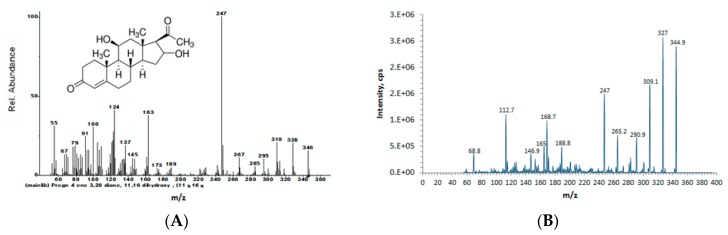
Relevant tandem mass (MS/MS) spectra. (**A**) 11, 16-Dihydroxypregn-4-ene-3, 20-dione (from NIST 14 mass spectral library); (**B**) isolated and purified compound (**3**).

The molecular formula of compound (**3**): C_21_H_30_O_4_, was identified with the MS/MS spectrum by searching the NIST14 mass spectral database (Figure 5).

#### 2.1.4. Compound (**4**): Tricin

**Figure 6:**
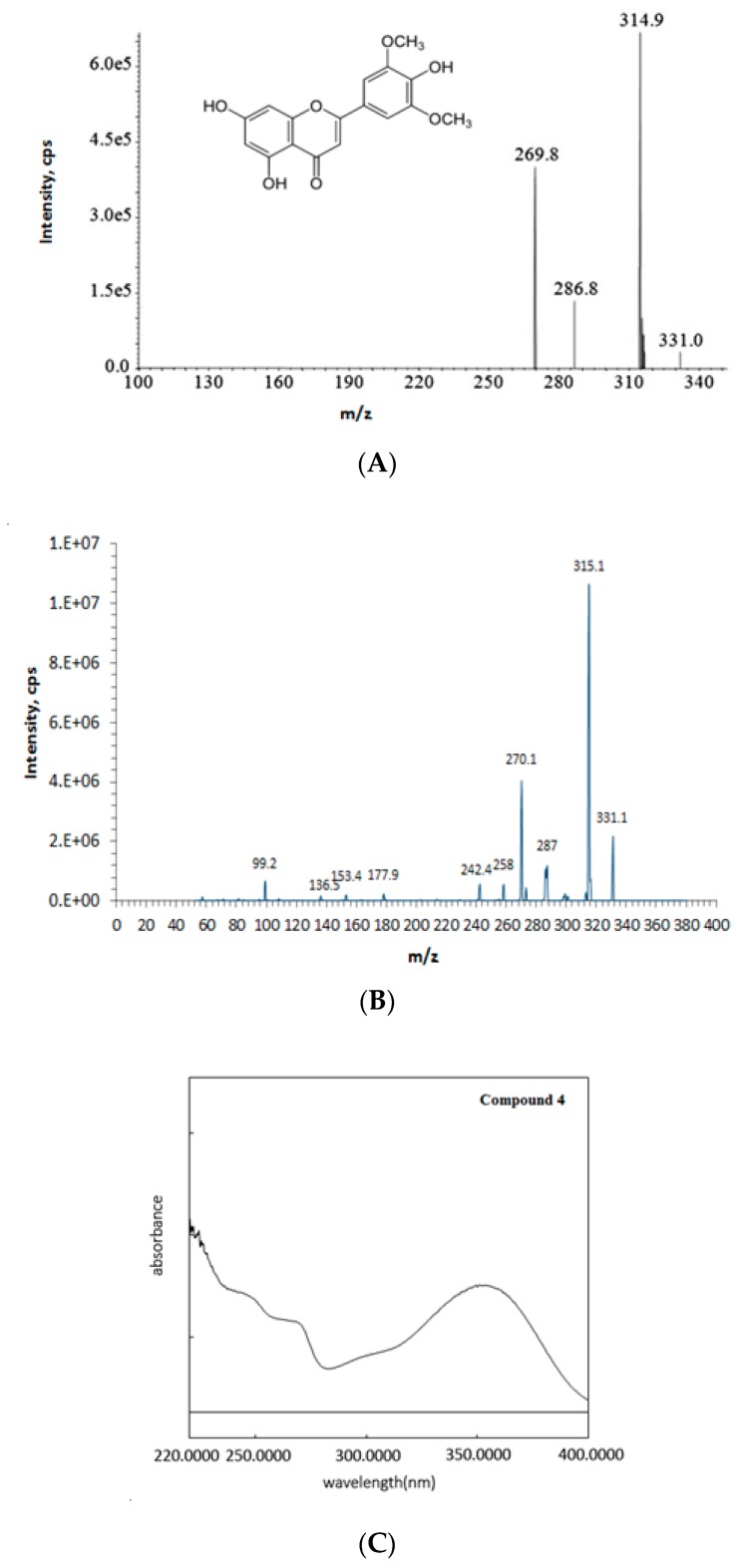
Relevant tandem mass (MS/MS) spectra. (**A**) Tricin (from literature [11]); (**B**) isolated and purified compound (**4**). (**C**) Compound (**4**) has absorption peaks (λ_max_) at 351 nm. A control of 100% methanol was used and auto-zeroed automatically by the software.

The molecular formula of compound (**4**), C_17_H_14_O_7_, was identified by comparing the MS/MS spectrum with the published literature [11] (Figure 6A,B). The UV spectrum (Figure 6C), obtained using λ_max_ at 351 nm, was consistent with the previous description [12].

**Figure 7:**
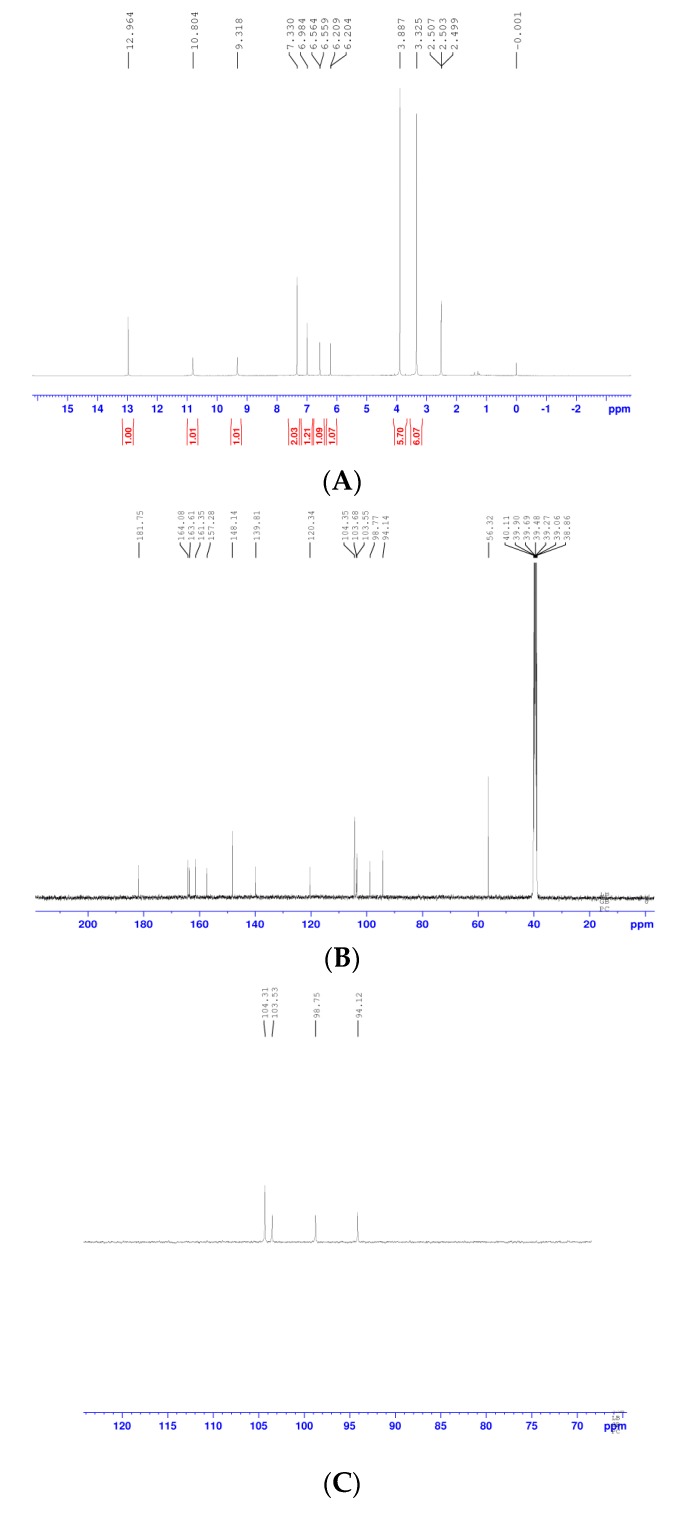

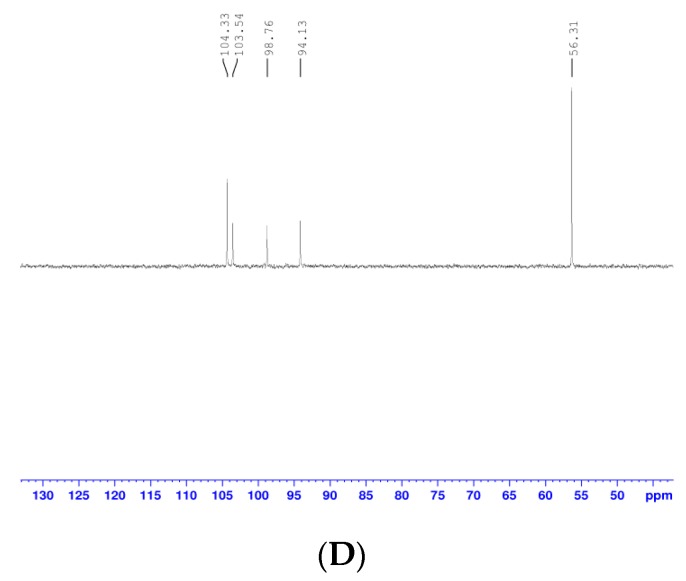
NMR spectra of Tricin. Purified Compound (**4**) in DMSO-*d*_6_ solution was conducted NMR analysis. ^1^H, ^13^C, DEPT90, and DEPT135-NMR spectra are shown in (**A**–**D**), respectively.

1H-NMR (400 MHz, DMSO-*d*_6_) δ (ppm): 12.964 (1H, s, 5-OH), 10.804 (s, 1H, 7-OH), 9.318 (s, 1H, 4-OH), 7.330 (2H, s, H-6′ and H-2′), 6.984 (1H, s, H-3), 6.564 (1H, d, *J* = 2.0 Hz, H-8), 6.209 (1H, d, *J* = 2.0 Hz, H-6), 3.887 (6H, s, 2OCH_3_). 13C-NMR (100 MHz, DMSO-*d*_6_) δ (ppm): 181.75 (C-4), 164.08 (C-2), 163.61 (C-7), 161.35 (C-5), 157.28 (C-9), 148.41 (C-3′ and C-5′), 139.81 (C-4′), 120.34 (C-1′), 104.35 (C-3), 103.68 (C-2′ and C-6′), 103.55 (C-10), 98.77 (C-6), 94.14 (C-8), 56.32 (2OCH_3_). DEPT90-NMR (DMSO-*d*_6_) δ (ppm): 94.12 (C-8), 98.75 (C-6), 103.53 (C-10), 104.31 (C-3). DEPT135-NMR (DMSO-*d*_6_) δ (ppm): 56.31 (2OCH_3_), 94.13 (C-8), 98.76 (C-6), 103.54 (C-10), 104.3 (C-3). DEPT spectra revealed that there were two primary carbons, five tertiary carbons, and ten quaternary carbons. A signal at δ3.887 (s, 6H) observed in the 1H-NMR spectrum and a signal at δ 56.32 observed in the 13C-NMR spectrum indicated that there were two equivalent methoxy groups. The NMR results (Figure 7) were consistent with the published data [13,14].

Compounds (**1**)–(**4**) identified by tandem mass spectrometry (MS^2^) are listed in Table 1. 

#### 2.1.5. Content of Analytes in IMP Aerial Part Ethyl Acetate Extract

Quantitative analysis of each isolated and purified compound in IMP aerial part ethyl acetate extract was determined by HPLC-DAD. The linearity of the calibration curve, limit of detection (LOD), and limit of quantification (LOQ) are listed in Table 2.

### 2.2. Growth Inhibitory Evaluation of Compounds *(**1**)–(**4**)* on Breast Cancer and Colorectal Cancer In Vitro

**Figure 8:**
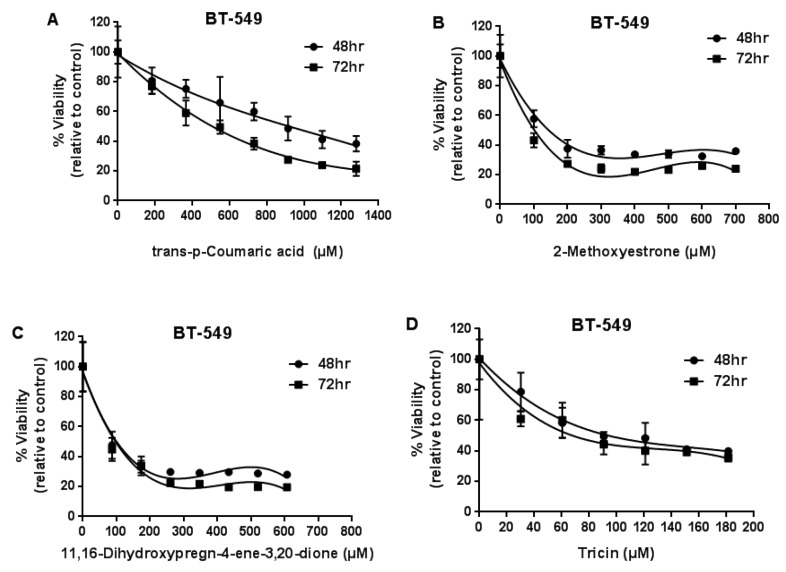
The growth inhibitory effects of compound (**1**)–(**4**) on BT-549 (breast cancer cell line) were evaluated after 48/72 h treatment by MTT assay.

The purified dried powder of each compound was dissolved in DMSO with a gradient of concentrations (µM). The growth inhibitory effects of compounds (**1**)–(**4**) on BT-549 (breast cancer cell line) were evaluated after 48/72 h treatment by MTT assay (Figure 8). Data are presented as mean values ±SD from three independent studies (*n* = 3). 

**Figure 9:**
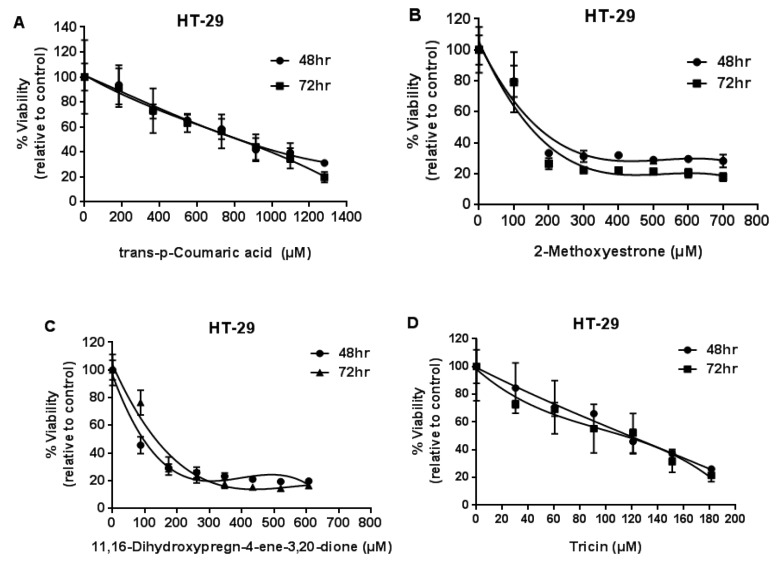
The growth inhibitory effects of compounds (**1**)–(**4**) on HT-29 (colon cancer cell line) were evaluated after 48/72 h treatment by MTT assay.

The growth inhibitory effects of compounds (**1**)–(**4**) on HT-29 (colon cancer cell line) are shown in Figure 9.

The half-maximal inhibitory concentration (IC50) of compounds (**2**), (**3**), and (**4**) on BT-549 breast cancer cell line (72 h) was 102, 97, and 68 µM, respectively. IC50 of compounds (**2**), (**3**), and (**4**) on a HT-29 colon cancer cell line (72 h) was 147, 134, and 114 µM, respectively. There were no statistically significant differences between 48 h and 72 h treatment groups (*p >* 0.05) (Table 3).

## 3. Discussion

A previous study showed that the 50% growth inhibitory effect (GI50) of the IMP aerial part ethyl acetate extract against HT-29 was 14.5 μg/mL [10]. The three isolated compounds, including 2-Methoxyestrone (**2**), 11, 16-Dihydroxypregn-4-ene-3, 20-dione (**3**), and Tricin (**4**), have considerable growth inhibitory activities on BT-549 and HT-29 with the IC50 values among 23–51 µg/mL. Synergy and positive interactions between isolated constituents may contribute to the greater effect of the crude extract against cancers that can be further investigated. 

Compound (**1**), trans-p-Coumaric acid, was able to induce apoptosis of HCT-15 colon cancer cells through a ROS-mitochondrial pathway with an IC 50 value of 1400 μM [15]. Natural trans-p-Coumaric acid exists in a wide variety of edible plants. The phenolic components from flaxseed oil was reported to have cytotoxic and pro-oxidant effects on MCF-7 human breast cancer cells [16]. The high gastric absorption efficiency of p-Coumaric acid was observed in rats, which makes it a potential bio-active compound in vivo [17]. Compound (**2**), 2-Methoxyestrone, is one kind of metabolite of estrone and estradiol. It is worth mentioning that 2-Methoxyestradiol was under a phase II clinical trial and expected to be a novel oral drug against multiple human melanoma, including breast cancer and ovarian cancer [18,19]. Metabolic inter-conversion between 2-Methoxyestrone and 2-Methoxyestradiol are based on the enzymatic catalyze reactions. Reductive activity promotes 2-Methoxyestrone conversion to 2-Methoxyestradiol. 2-Methoxyestrone can be formed by the enzymatic oxidation of 2-Methoxyestradiol [20]. Our study first reported the growth inhibitory activities of compound (**3**), 11, 16-Dihydroxypregn-4-ene-3, 20-dione, against BT-549 and HT-29 cancer cell lines. The structure of 11, 16-Dihydroxypregn-4-ene-3, 20-dione is similar to the well-known endogenous steroid (11α-Hydroxyprogesterone, C_21_H_30_O_3_). Transformations of 11α-Hydroxyprogesterone generate a series of metabolites. Amounts of metabolites with different isoforms were identified as novel candidates of steroid drugs [21]. The molecular mechanisms of 11, 16-Dihydroxypregn-4-ene-3, 20-dione against cancers can be further investigated. Compound (**4**), Tricin, a well-studied bio-active flavonoid, is widely distributed in rice bran and bamboo leaves [22,23]. A previous study also isolated Tricin from the aerial part of *Imperata cylindrica* (L.) Beauv. [5]. Tricin was reported to have remarkable anti-cancer potential against SW-480 colon cancer cells and MDA-MB-468 breast cancer cells, and is safe for clinical development as a cancer preventive agent [24,25,26,27,28].

## 4. Materials and Methods 

### 4.1. Cells, Chemicals and Reagents 

BT-549 and HT-29 cell lines were obtained from ATCC (Manassas, VA, USA). BT-549 and HT-29 cells were cultured at 37 °C in a humidified atmosphere of 5% CO_2_ in RPMI 1640 (Gibco, Carlsbad, CA, USA) supplemented with 10% fetal bovine serum (FBS) (Gibco, Carlsbad, CA, USA). Acetonitrile (ACN) (E. Merck, Darmstadt, Germany), Methanol (E. Merck, Darmstadt, Germany) and trifluoroacetic acid (TFA) (Sigma Aldrich, St. Louis, MO, USA) were of HPLC grade, and distilled and deionized water (ddH_2_O) was prepared using a Millipore water purification system (Millipore, Milford, MA, USA). All other reagents used in this study were of analytical reagent grade or higher and purchased from Sigma Aldrich.

### 4.2. Preparation of Powder Extract of IMP Aerial Part

The extraction method was described previously [10].

### 4.3. HPLC Analysis

The HPLC fingerprint was analyzed on a HP1100 series system (Santa Clara, CA, USA) equipped with a diode-array detector. An extract solution of 50 mg/mL (dissolved in methanol) was filtered with a 0.22 μm polytetrafluoroethylene (PTFE) membrane. A 15 μL sample was injected to a semi-preparative HPLC column (ALLTIMA C18, 5 μm, 250 mm × 10 mm i.d. Hichrom, Searle, UK) and detected at 323 nm. The initial mobile phase composed of solvent A (0.1% TFA in ddH_2_O) and solvent B (100% methanol). The gradient for the HPLC analysis was programmed as follows: 0–5 min, 65% B; 5–15 min, 70% B; at a flow rate of 1.5 mL/min; 15–25 min, 80% B, at a flow rate of 1.0 mL/min; 25–40 min, 85% B, at a flow rate of 0.8 mL/min; 40–50 min, 100% B, at a flow rate of 2.0 mL/min, and then was held for additional 5 min.

### 4.4. Isolation and Purification of Compounds *(**1**)–(**4**)* by HPLC

Fractions were collected manually by observing the elution profile of the chromatography workstation. The elution profile was programmed with the gradient mobile phase composed of solvent A (0.1% TFA in ddH_2_O), solvent B (100% methanol), and solvent C (100% ACN). Fractions were isolated and purified with the semi-preparative HPLC column (ALLTIMA C18, 5 μm, 250 mm × 10 mm i.d. Hichrom, Searle, UK). The gradients used for collecting each fraction were set as follows: Fraction (1), 0–14 min, 60% C; 14–19 min, 100%; at a flow rate of 1.5 mL/min; Fractions (2) and (3), 0–15 min, 85% B; 15–18 min, 100% B; at a flow rate of 2.0 mL/min; Fraction (4), 0–15 min, 85% B; at a flow rate of 2.0 mL/min; and 15–17 min, 100% B; at a flow rate of 2.5 mL/min. The purity of each HPLC fraction was calculated based on the proportion of the target peak area. The purified HPLC eluent was lyophilized and stored at −20 °C for further use.

### 4.5. Mass Spectrometry

The identification of each purified component was performed on a tandem mass spectrometer equipped with an electrospray ionization source. Each purified compound was dissolved in methanol at an appropriate concentration and was infused into the QTRAP 5500 mass spectrometer system (AB SCIEX, Framingham, MA, USA) equipped with a Turbo VTM Spray ion source. Multiple reaction monitoring (MRM) in both positive and negative mode was used to enhance the selectivity of detection. The source-dependent parameters for the mass spectrometer (MS) were set as follows: ion spray voltage (IS) = ±5500 V; curtain gas (CUR) = 20 psi; collision gas (CAD) = 10 psi; nebulizer gas (GS1) = 12 psi, heater gas (GS2) = 0 psi, and source temperature (TEM) = 0 °C. The fraction-dependent parameters were set as follows: declustering potential (DP) = +120.0 V/−130.0 V; entrance potential (EP) = ±10.0 V; collision cell exit potential (CXP) = ±13.0 V. The MS/MS optimized collision energy applied to compounds (**1**)–(**4**) was given as follows: 25 V in positive mode, 25 V in negative mode, 25 V in negative mode, and 40 V in positive mode, respectively. For trans-p-Coumaric acid standard, the collision energy applied was 10 V in positive mode. Raw data and images of spectra were generated by Analyst^®^ Software (Redwood, CA, USA) and modified using Excel^®^ (Redmond, WA, USA).

### 4.6. Ultraviolet-Visible Spectrophotometry

The UV spectrum of compound (**4**) was measured using a Shimadzu UV-3600 spectrophotometer (Shimadzu Corporation, Kyoto, Japan). Each absorption spectrum was recorded from 200.00 nm to 400.00 nm. Profiles were generated by UVProbe 2.21 Software (Shimadzu Corporation, Kyoto, Japan). A control of 100% methanol was set and auto-zeroed automatically by software. 

### 4.7. NMR Analysis

A 5 mg sample of purified compound (**4**) was dissolved in DMSO-*d*_6_, and 1H NMR (400 MHz), 13C NMR (100 MHz), DEPT 90, DEPT 135 spectra were recorded using the Bruker Avance III 400 MHz NMR spectrometer spectroscopy (Bruker Corporation, Solna, Sweden). All chemical shifts were reported in δ (ppm) relative to tetramethylsilane (TMS).

### 4.8. Quantitative Analysis

The content of each identified compound in IMP aerial part ethyl acetate extract was determined using a HPLC-DAD system. The linearity and range was evaluated by constructing a calibration curve (peak area vs concentration). Quantification was performed upon six levels of external standards. The limit of detection (LOD) was determined as the concentration with a signal-to-noise ratio of three, and the limit of quantification (LOQ) was determined as the concentration with a signal-to-noise ratio of ten.

### 4.9. MTT Assay

The growth inhibitory effects of compounds (**1**)–(**4**) on HT-29 (colon) and BT-549 (breast) cancer cell lines were evaluated. Cells were seeded at 4 × 10^3^ cells per 96-well and incubated for 24 h. The cells were then treated by 0.5% DMSO (as solvent control) or various concentrations of compounds (as treatment group) and incubated at 37 °C for 48 and 72 h. The MTT assay and data analysis were performed as previously described [29].

### 4.10. Data Analysis

All statistics were calculated with SPSS 17.0 software and data were expressed as mean ± standard deviation (SD) for each analyte. For MS/MS spectrometry analysis, each mass spectrum shown was the average spectra of each sample detected with ten repetitions in each analysis. Compounds were identified by comparing the tandem mass (MS/MS) fragmentation patterns with those in the literatures, NIST14 mass spectral database, and the MS Search Program v.2.2 (National Institute of Standards and Technology, Gaithersburg, MD, USA). For the viability assay, a nonlinear regression test was applied to obtain a fit curve (R^2^ > 0.98). Analysis of the differences between the 48/72 h treatment groups was carried out by one-way ANOVA (coupled with a post-test, Dunnett’s test) with * *p* < 0.05.

## 5. Conclusions

In this study, it is the first time that trans-p-Coumaric acid (**1**); 2-Methoxyestrone (**2**); 11, 16-Dihydroxypregn-4-ene-3, 20-dione (**3**), and Tricin (**4**) were isolated and identified from IMP aerial part ethyl acetate extract. 2-Methoxyestrone, 11, 16-Dihydroxypregn-4-ene-3, 20-dione and Tricin possess considerable growth inhibitory activities against BT-549 breast and HT-29 colon cancer cell lines. The data provided important information about the bio-active components from IMP aerial part ethyl acetate extract. *Imperata cylindrica* (L.) Raeusch., one kind of traditional herbal medicine, has rational medical application potentials with respect to breast and colorectal cancer prevention.

## Figures and Tables

**Table 1:** Characteristics of compound (**1**)–(**4**) identified by tandem mass spectrometry.

Analyte	Ion Mode	Molecular Formula	CAS No.	MS/MS Fragments (*m*/*z*)
trans-p-Coumaric acid	[M+H]^+^	C_9_H_8_O_3_	501-98-4	165.7, 147.4, 136.3, 118.9, 90.5
2-Methoxyestrone	[M-H]^−^	C_19_H_24_O_3_	362-08-3	299.9, 285.0, 271.8, 256.3, 243.8
11, 16-Dihydroxypregn-4-ene-3, 20-dione	[M-H]^−^	C_21_H_30_O_4_	55622-61-2	344.9, 327.0, 309.1, 290.9, 265.2, 247.0
Tricin	[M+H]^+^	C_17_H_14_O_7_	520-32-1	331.1, 315.0, 287.0, 270.1, 258.0, 242.4

**Table 2:** Content of analytes in IMP aerial part ethyl acetate extract.

Analytes	Calibration Curves ^a^	R^2 b^	Linear Range (mg/mL)	LOD ^c^ (µg/mL)	LOQ ^d^ (µg/mL)	Contents of Analytes (mg/g Extract, *n* = 3)
trans-p-Coumaric acid	y = 114751x − 31.91	0.9999	0.0010–0.25	0.30	0.95	0.12 ± 0.010
2-Methoxyestrone	y = 13217x + 195.65	0.9992	0.031–1.00	7.28	24.84	0.86 ± 0.042
11,16-Dihydroxypregn-4-ene-3, 20-dione	y = 15063x + 1173.70	0.9995	0.12–2.50	6.71	22.91	0.65 ± 0.13
Tricin	y = 35025x − 126.29	0.9999	0.016–2.00	3.23	11.02	0.59 ± 0.041

^a^ y, the value of peak area (by HPLC-DAD at 323 nm); x, the value of concentration (mg/mL); ^b^ R^2^, correlation coefficient for six points on the calibration curves (*n* = 3); ^c^ LOD, limit of detection (S/N = 3); ^d^ LOQ, limit of quantification (S/N = 10).

**Table 3:** IC50 of compounds (**1**)–(**4**) on BT-549 and HT-29 cancer cell lines.

Cancer Unit		Treatment Group (48/72 h)			
		Trans-p-Coumaric Acid	2-Methoxyestrone	11,16-Dihydroxypregn-4-ene-3, 20-dione	Tricin
BT-549	µg/mL	151/83	43/31	35/34	31/23
	µM	920/507	144/102 ^a^	101/97 ^a^	95/68 ^a^
HT-29	µg/mL	135/135	51/44	33/46	39/38
	µM	821/821	169/147 ^a^	96/134 ^a^	118/114 ^a^

^a^ Numbers identified refer to the considerable growth inhibitory activity with half-maximal inhibitory concentration (IC50 < 150 µM).

## Abbreviations

HPLC-DAD, high-performance liquid chromatography–diode array detector (HPLC-DAD); ESI-MS/MS, electrospray ionization tandem mass spectrometry; NMR, nuclear magnetic resonance; IMP, Imperata cylindrica; MTT, 3-(4,5-Dimethylthiazol-2-yl)-2,5-Diphenyltetrazolium Bromide; ACN, acetonitrile; TFA, trifluoroacetic acid; λmax, wavelength of maximum absorption; LOD, limit of detection; LOQ, limit of quantification; IC50, half-maximal inhibitory concentration; TMS, tetramethylsilane; SD, standard deviation.
